# Long-Term Health Outcomes of Regular, Moderate Red Wine Consumption

**DOI:** 10.7759/cureus.46786

**Published:** 2023-10-10

**Authors:** Jeffrey S Wojtowicz

**Affiliations:** 1 Pharmacology, Tutela Pharmaceuticals, Boston, USA

**Keywords:** long term, alcohol, health outcomes, moderation, red wine

## Abstract

Studies that are conducted to assess alcohol’s long-term health outcomes generally report the results as a pooled analysis across all types of alcohol. Questions have been raised regarding potential health differences between types of alcohol, such as beer, wine, or spirits. While these three share the same alcohol in the form of ethanol, they differ in the other compounds they contain that are particular to each type of alcohol, specifically the polyphenols in red wine. The generalizability of pooled results may be limited due to the differences in health outcomes that may exist between different types of alcohol and lead to overall conclusions that differ from the subset analysis by type of alcohol that is often reported in the data tables of an article.

The objective of this systematic review was to specifically address the assessment of the long-term health outcomes of regular, moderate, red wine consumption. PubMed was searched from 1987 through June 2023. Studies were included if they met all the following criteria: adult participants, red wine consumption and its frequency (close to daily), volume in moderation (1 glass/day for women, 2 glasses/day for men), and measurement of long-term (> 2 years) health outcomes. Nonclinical animal studies, or studies with an endpoint as a marker or biomarker, without a health outcome, of short duration (< 2 years), small size (< 25 subjects), a focus on binge drinking, no wine analysis performed, review articles, meta-analysis, or editorial/commentary were excluded.

A total of 74 studies met the inclusion/exclusion criteria. Of these, 27 (36%) evaluated cancer outcomes, 14 (19%) evaluated cardiovascular outcomes, 10 (14%) evaluated mortality, 7 (9%) evaluated weight gain, 5 (7%) evaluated dementia, and the remaining 11 evaluated a variety of health outcomes. There were no studies that demonstrated an association between red wine consumption and negative health outcomes. Forty-seven studies demonstrated an association between red wine consumption and positive health outcomes, whereas 26 studies were neutral, and one had mixed results where women had a positive health outcome and men were neutral. All studies on mortality and dementia showed positive health outcomes.

From this systematic review of the literature, there is no evidence of an association between moderate red wine consumption and negative health outcomes. Across the various outcomes assessed, a beneficial effect of moderate red wine consumption was consistently seen for mortality and dementia, along with certain cancers (e.g., non-Hodgkin lymphoma) and cardiovascular conditions (e.g., metabolic syndrome). For other health outcomes, the association was neutral, i.e., neither harmful nor beneficial.

This review is not intended to encourage red wine consumption for health outcomes but rather to avoid discouraging moderate red wine consumption based on misunderstanding or misinterpretation of the red wine data due to the reporting of pooled data across all types of alcohol.

## Introduction and background

Many studies have been conducted that look at the effect alcohol consumption has on health outcomes. Studies generally report the results as a pooled analysis across all types of alcohol. However, queries regarding the potential health benefits of the different types of alcohol, such as beer, wine, or spirits, have been raised. While these three may have the same alcohol in the form of ethanol in common, they differ in the other compounds they contain. Notably, since the reporting of the French paradox [1], questions have been raised regarding differences in health outcomes based on the type of alcohol consumed, especially positive health outcomes from red wine consumption. It has been surmised that the polyphenols present in red wine may have a positive influence on health outcomes [2]. Pooled results may have limited generalizability due to the differences in health outcomes between different types of alcohol. Moreover, pooled results lead to overall conclusions that differ from the subset analysis by type of alcohol, which is often reported in the data tables of a study. The objective of this systematic review was to specifically address the assessment of the long-term health outcomes of regular, moderate, red wine consumption.

## Review

Inclusion and exclusion criteria

**Figure 1:**
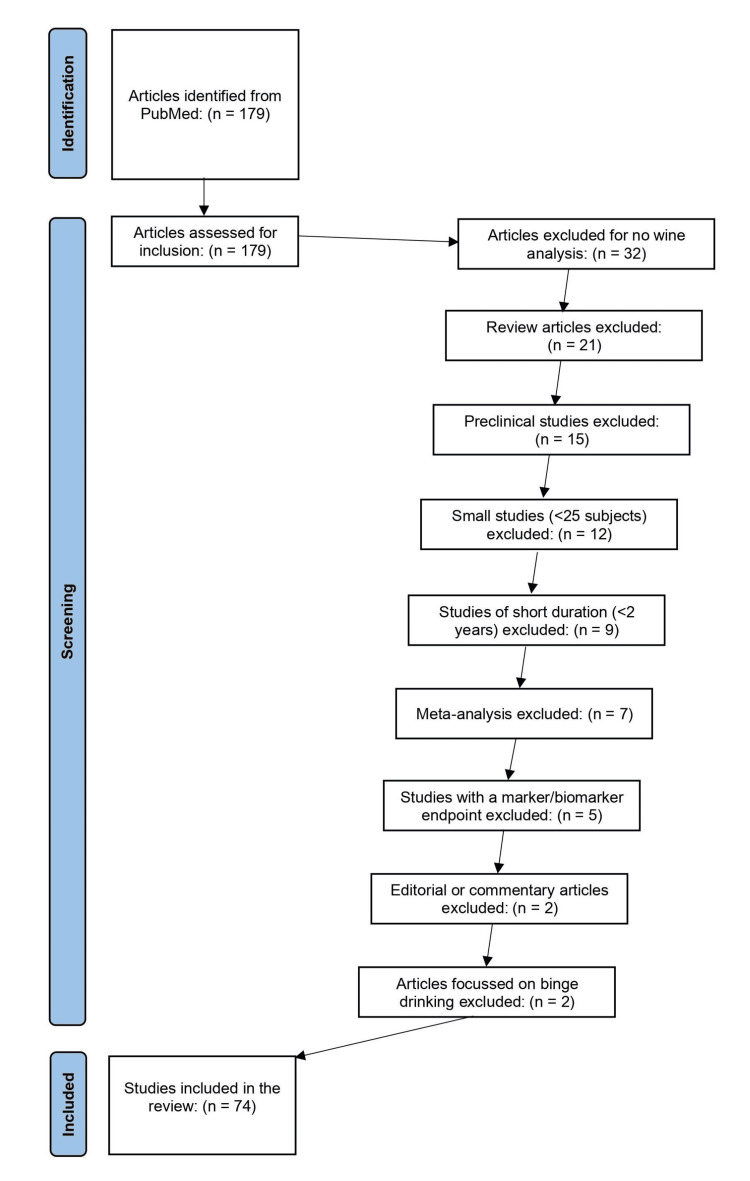
Literature search

To conduct this review, PubMed was searched from 1987 through June 2023. Studies were included if they met all of the following criteria: adult participants, the consumption of red wine, its frequency (close to daily), volume in moderation (1 glass/day for women, 2 glasses/day for men), and measurement of its long-term (> 2 years) health outcomes. The following studies were excluded: nonclinical animal studies, endpoint as a marker/biomarker and not a health outcome, short duration (< 2 years), small size (< 25 subjects), focus on binge drinking, no wine analysis performed, review articles, meta-analysis, or editorial/commentary. From this search, 74 studies met the full criteria (Figure 1).

In this review, a common definition for moderate consumption was used, and study amounts were converted for ease of comparison. Moderate red wine consumption is defined as 1 drink = 1 glass = 5 oz = ~14 grams of alcohol; 1 glass/day for women, 2 glasses/day for men [3,4].

Results

A total of 74 studies met the inclusion criteria. Of these, 27 (36%) evaluated cancer outcomes, 14 (19%) evaluated cardiovascular outcomes, 10 (14%) evaluated mortality, 7 (9%) evaluated weight gain, 5 (7%) evaluated dementia, and the remaining 11 dealt with a variety of health outcomes. There were no studies that demonstrated an association between red wine consumption and negative health outcomes. Forty-seven studies demonstrated an association between red wine consumption and positive health outcomes, 26 studies were neutral, and one had mixed results where women had a positive health outcome and men were neutral. All studies on mortality and dementia showed positive health outcomes. Table 1 shows the breakdown of moderate red wine consumption on specific health outcomes.

**Table 1:** Red wine health outcomes by disease * Positive for women, neutral for men **Conditions included age-related macular degeneration, amyotrophic lateral sclerosis, common cold, COVID-19, depression, erosive esophagitis, kidney stones, liver cirrhosis, multiple sclerosis, pulmonary function, and rheumatoid arthritis

Disease	# of Studies	Positive Health Outcomes	Neutral Health Outcomes	Mixed* Health Outcomes	Negative Health Outcomes
Cancer	27	9	17	1	0
Cardiovascular	14	10	4	0	0
Mortality	10	10	0	0	0
Weight Gain	7	4	3	0	0
Dementia	5	5	0	0	0
Multiple Conditions**	11	9	2	0	0
Totals	74	47	26	1	0

A detailed summary of the health outcomes associated with moderate red wine consumption from all 74 studies can be found in Table 2.

**Table 2:** Summary of the health outcomes associated with moderate red wine consumption RR: Relative risk; OR: Odds ratio; HR: Hazard ratio; HDL-C: High-density lipoprotein cholesterol; HDL: High-density lipoprotein; HOMA-insulin resistance: Homeostatic model assessment of insulin resistance; BP: Blood pressure; CV: Cardiovascular; FEV1: Forced expiratory volume in 1 second; FVC: Forced vital capacity; SE: Standard error

Organ / System / Disease	Subsection	Gender	Health Outcome	Moderate Wine Consumption Drink = Glass = 5 oz (~14gm of alcohol) 1/day for women, 2/day for men	Relevant Findings	Results
Arthritis	Rheumatoid Arthritis [5]	Women	Positive outcome	> 4 glasses of wine (red or white) per week	Moderate wine consumption was associated with a 37% decrease in the risk of developing rheumatoid arthritis.	In this study of 34,141 women, moderate wine consumption was associated with a 37% decrease in the risk of developing rheumatoid arthritis versus non-drinkers (RR = 0.63 (0.42-0.96) P = 0.04). Among non-smokers, the risk decreased even more (RR = 0.38 (0.15-0.97)). There was no separate analysis for red wine.
Cancer	Breast Density [6]	Women	Positive outcome	0.5 to 1 glass of red wine per day	Moderate red wine consumption was associated with decreased breast density in postmenopausal women.	In this study of 1508 women with a positive family history of breast cancer, moderate red wine consumption was associated with decreased breast density as a percentage of breast tissue in postmenopausal women versus non-drinkers by 18% (RR = 0.82 (0.71-0.97) P-trend = 0.02). There was no effect of moderate red wine consumption on breast density for premenopausal women.
Cancer	Breast Cancer [7]	Women	Positive outcome	1 glass of red wine per day	Moderate lifetime red wine consumption had a consistent inverse association with mammographic density in premenopausal women.	In this study of 166 women (average age 42) followed from birth, there was a consistent, statistically significant, inverse association between moderate lifetime red wine intake and mammographic density in these premenopausal women versus non-drinkers (r = 0.73; P < 0.0001). This reduction in risk was not seen with white wine or other alcoholic beverage types.
Cancer	Breast Cancer [8]	Women	Neutral outcome	0.5 to 1.5 glasses of red wine per day	Moderate red wine consumption was not associated with a risk of breast cancer.	In this study of 13,885 women (age 20 to 69), moderate red wine consumption was not associated with the risk of breast cancer versus non-drinkers (OR = 1.17 (0.9-1.51)).
Cancer	Basal Cell Carcinoma (BCC) [9]	Men and women	Positive outcome for women, neutral outcome for men	> 1 glass of red wine per day	Moderate red wine consumption was associated with a reduced risk of BCC for women but did not have any effect on the risk for men. NOTE: Moderate white wine had an increased risk of BCC for women but no effect on the risk for men.	In this study of 107,975 men and women followed for eight to 10 years, moderate red wine consumption was associated with a reduced risk of BCC for women versus non-drinkers (RR = 0.56 (0.29-1.08) P-trend = 0.004), but did not have any effect on risk for men versus non-drinkers (RR = 1.0 (0.67-1.49) P-trend = 0.64). NOTE: Moderate white wine had an increased risk of BCC for women versus non-drinkers (RR = 1.39 (1.11-1.73) P-trend = 0.0002), but no effect on risk for men.
Cancer	Basal Cell Carcinoma (BCC) [10]	Men and women	Neutral outcome	> 1 glass of red wine per day	Moderate red wine consumption was not associated with BCC risk. NOTE: Moderate white wine consumption was associated with BCC risk.	In this study of 211,462 men and women (age 36 to 55) followed for 16+ years, moderate red wine consumption was not associated with BCC risk versus non-drinkers (HR = 0.99 (0.89-1.1) P = 0.67). NOTE: Moderate white wine consumption was associated with BCC risk (HR = 1.22 (1.06-1.4) P<0.0001).
Cancer	Basal Cell Carcinoma (BCC) [11]	Men and women	Neutral outcome	Average of 1 glass of red wine per day	Moderate red wine consumption was not associated with BCC risk.	In this study of 380 men and women (age <40, average 36) who were diagnosed with BCC, there was no association between red wine consumption and BCC, either below 1 glass per day (OR = 1.07 (0.66-1.75) P-trend = 0.341) or above 1 glass per day (OR = 1.07 (0.65-1.76) P-trend = 0.341) versus non-drinkers who were matched controls.
Cancer	Melanoma [12]	Men and women	Neutral outcome	2 glasses of red wine per day	Moderate red wine consumption was not associated with a risk of melanoma. NOTE: Moderate white wine consumption did increase the risk of melanoma.	In this study of 210,252 men and women (average age 50) followed for 18 years, moderate red wine consumption was not associated with a risk of melanoma versus non-drinkers (HR = 1.01 (0.77-1.32) P = 0.47). NOTE: Moderate white wine consumption did have an increased risk of melanoma versus non-drinkers (HR = 1.42 (1.13-1.80) P < 0.01).
Cancer	Bladder Cancer [13]	Men and women	Neutral outcome	1 glass of wine (red or white) per day	Moderate wine consumption was not associated with the risk of bladder cancer.	In this study of 476,160 men and women (average age of 51) followed for 14 years, moderate wine consumption was not associated with the risk of bladder cancer versus non-drinkers (HR = 1.02 (0.97-1.07)). There was no separate analysis for red wine.
Cancer	Colon Cancer [14]	Men and women	Positive outcome	Up to 1 glass of red wine per day	Moderate red wine consumption in subjects with resected stage III colon cancer was associated with improved survival and reduced recurrence of colon cancer.	In this study, 1,925 men and women with resected stage III colon cancer were followed for an average of 6.5 years and consumed red wine in moderation. Disease-free survival, overall survival, and timeyo recurrence improved versus non-drinkers (DFS HR = 0.82 (0.69-0.98) P = 0.031; OS HR = 0.8 (0.66-0.98) P = 0.027; TTR HR = 0.83 (0.69-1.0) P = 0.049).
Cancer	Colorectal Cancer [15]	Men and women	Neutral outcome	1 to 2 glasses of wine (red or white) per day	Moderate wine consumption was not associated with the risk of colorectal cancer.	In this study of 190,698 men and women (age 45 to 75) followed for an average of 17 years, moderate wine consumption was not associated with the risk of colorectal cancer versus non-drinkers (HR = 1.22 (0.85-1.76) P-trend = 0.02). There was no separate analysis for red wine.
Cancer	Colon and Rectal Cancer [16]	Men and women	Neutral outcome	> 1.5 glasses of wine (red or white) per day	Moderate wine consumption demonstrated no increase in the risk of colon or rectal cancer.	In this study of 29,132 men and women with a mean follow-up of 14.7 years, moderate wine consumption demonstrated no increase in the risk of colon cancer (RR = 0.5 (0.2-1.0) P-trend = 0.07) or rectal cancer (RR = 0.9 (0.4-2.1) P-trend = 0.87) versus non-drinkers. There was no separate analysis for red wine.
Cancer	Rectal Cancer [17]	Men and women	Neutral outcome	1 glass of red wine per day for women and 2 glasses per day for men	Moderate red wine consumption was associated with a non-statistically significant reduced risk of a TP53 mutation (marker for rectal cancer).	In this study of 750 men and women with rectal cancers, using a 20-year follow-up, the authors observed a non-statistically significant reduced risk of a TP53 mutation (marker for rectal cancer) among those who drank red wine versus non-drinkers or non-red wine drinkers (OR = 0.64 (0.35-1.18) P-trend = 0.39).
Cancer	Gastric Cancer [18]	Men and women	Neutral outcome	> 2 glasses of wine (red or white) per day	Moderate wine consumption was not associated with gastric cancer risk.	In this study of 478,459 men and women followed for about 15 years, moderate wine consumption was not associated with gastric cancer risk versus very light drinkers (i.e., < 1/3 glass per day) (HR = 0.89 (0.6-1.3) P-trend = 0.7). There was no separate analysis for red wine.
Cancer	Barrett's Esophagus (BE) and Esophageal Adenocarcinoma (EAC) [19]	Men and women	Neutral outcome	1 glass of wine (red or white) per day	Moderate wine consumption was not associated with the risk of BE or EAC.	In this study of 24,068 men and women (age 39 to 79) followed for 12 years, moderate wine consumption was not associated with the risk of BE or EAC versus non-drinkers (HR = 0.49 (0.23-1.04) P = 0.06). There was no separate analysis for red wine.
Cancer	Head and Neck Cancer [20]	Men and women	Neutral outcome	Up to 1.5 glasses of wine (red or white) per day	Moderate consumption of wine had no association with the risk of head and neck cancer.	In this study of 4288 men and women (average age 61) followed for 17+ years, moderate consumption of wine had no association with the risk of head and neck cancer versus non-drinkers (HR = 0.95 (0.63-1.44)). There was no separate analysis for red wine.
Cancer	Laryngeal Cancer [21]	Men	Neutral outcome	1 to 5 glasses of wine (red or white) per day	Moderate wine consumption in men who are smokers was associated with no greater risk of laryngeal cancer.	In this small study of 107 men who had laryngeal cancer and were smokers (age 30 to 89) plus 290 matched controls, moderate wine consumption was associated with no greater risk of laryngeal cancer versus non-drinkers (OR = 0.8 (0.5-1.5)). There was no separate analysis for red wine.
Cancer	Lung Cancer [22]	Men	Positive outcome	Average of 2.5 glasses of red wine per day	Moderate red wine consumption among smokers was associated with a 60% reduced risk of lung cancer.	In this study of 78,168 men (age 45 to 69), among smokers, moderate red wine consumption was associated with an approximately 60% reduced risk of lung cancer versus non-drinkers (HR = 0.39 (0.14-1.08) P-trend = 0.03).
Cancer	Lung Cancer [23]	Men and women	Positive outcome	Average of 3.2 glasses of red wine per day	Moderate red wine consumption was associated with a decrease in the risk of developing lung cancer.	In this study of 319 men and women (including those with lung cancer and matched controls), moderate red wine consumption was associated with a decrease in the risk of developing lung cancer versus non-drinkers (OR = 0.43 (0.19-0.96)).
Cancer	Non-Hodgkin Lymphoma (NHL) [24]	Women	Positive outcome	> 1 glass of red wine per day	Moderate red wine consumption was associated with a decreased risk of NHL.	In this study of 35,156 women (age 55–69) during the nine-year follow-up, moderate red wine consumption was associated with a decreased risk of NHL compared to non-drinkers (RR = 0.21 (0.05–0.86) P-trend = 0.02).
Cancer	Non-Hodgkin Lymphoma (NHL) [25]	Men and women	Positive outcome	> 1 glass of wine (red or white) per day	Moderate wine consumption was associated with a lower risk of NHL among men and women.	In this large prospective study of 143,124 men and women (age 50 to 74) followed for 15 years, moderate wine intake of > 1 drink/day was associated with a 45% lower risk of NHL versus non-drinkers (RR = 0.60 (0.37-0.95)). There was no separate analysis for red wine.
Cancer	Prostate Cancer [26]	Men	Positive outcome	1 to 2 glasses of red wine per day	Moderate red wine intake after prostate cancer diagnosis was associated with a lower risk of death and a lower risk of progression to lethal disease. Cancer-free men who consumed red wine had a slightly lower risk of lethal prostate cancer.	In this study of 47,568 men followed over 26 years, 5182 developed prostate cancer and among those with a prostate cancer diagnosis; moderate red wine consumption versus abstainers was associated with a lower risk of progression to lethal prostate cancer (HR = 0.50 (0.29-0.86) P-trend = 0.05) and was also associated with a lower risk of death (HR= 0.74 (0.57-0.97) P-trend = 0.007). Cancer-free men who consumed red wine had a slightly lower risk of lethal prostate cancer compared with abstainers.
Cancer	Prostate Cancer [27]	Men	Positive outcome	> 1 glass of red wine per day	Moderate red wine consumption was associated with a decreased risk of prostate cancer.	In this study of 1456 men (age 40 to 64), moderate red wine consumption was associated with a decreased risk of prostate cancer versus non-drinkers (OR = 0.45 (0.23-0.85) P-trend = 0.02). For each additional glass of red wine consumed per week, there is a 6% decrease in the RR of prostate cancer (OR = 0.94 (0.9-0.98) P-trend = 0.02).
Cancer	Prostate Cancer [28]	Men	Neutral outcome	1 to 2.5 glasses of red wine per day	Moderate red wine consumption had no association with prostate cancer risk.	This study included 65,972 men (age 45 to 69) with 304,484 person-years of follow-up. Moderate consumption of red wine had no effect on the risk of prostate cancer versus non-drinkers (HR = 0.88 (0.70–1.12) P-trend = 0.92).
Cancer	Prostate Cancer [29]	Men	Neutral outcome	> 1 glass of red wine per day	Moderate consumption of red wine daily was not associated with a risk of prostate cancer.	In this study of 58,279 men (age 55 to 69) followed for 6.3 years, moderate consumption of red wine daily was not associated with a risk of prostate cancer versus non-drinkers (RR = 0.8 (0.4-1.7) P-trend = 1.00).
Cancer	Prostate Cancer [30]	Men	Neutral outcome	> 1 glass of red wine per day	Moderate red wine consumption had no association with prostate cancer risk.	In this study of 45,433 men (age 40 to 75) followed for 16 years, red wine consumption had no effect on the risk of prostate cancer versus non-red wine drinkers (RR = 1.06 (0.72-1.56) P-trend = 0.57).
Cancer	Prostate Cancer [31]	Men	Neutral outcome	Up to 2 glasses of red wine per day	Moderate red wine consumption had no association with prostate cancer risk.	In this study of 34,565 men (age 50 to 76), moderate red wine consumption had no association with prostate cancer risk versus non-drinkers (HR = 0.98 (0.72-1.33) P-trend = 0.23).
Cancer	Thyroid Cancer [32]	Men and women	Neutral outcome	> 1 glass of wine (red or white) per day	Moderate consumption of wine was not associated with a risk of thyroid cancer.	In this study of 490,159 men and women (age 50 to 71) followed over 7.5 years, moderate consumption of wine was not associated with a risk of thyroid cancer versus non-drinkers (RR = 0.76 (0.45-1.29) P-trend = 0.69). There was no separate analysis for red wine.
Cardiovascular	Cardiometabolic Syndrome [33]	Men and women	Positive outcome	1 glass of red wine per day with dinner	Moderate red wine consumption with dinner demonstrated improved metabolic syndrome criteria.	In this Randomized Controlled Trial of 224 men and women with well-controlled Type 2 Diabetes Mellitus who were non-drinkers, participants were randomly assigned to 5oz of water, white wine, or red wine with dinner for 2 years. Red wine consumption demonstrated improved metabolic syndrome criteria versus water drinkers (decrease in # of components of the metabolic syndrome by 0.34 (-0.68 to -0.001) P = 0.049). Statistically significant improvements were seen in HDL-C, apolipoprotein (a), and total cholesterol/HDL ratio for red wine drinkers versus water drinkers or white wine drinkers. Non-statistically significant improvements were seen in Fasting Plasma Glucose and HOMA-Insulin Resistance for red wine drinkers versus water drinkers. NOTE: Sleep quality also improved for red wine drinkers versus water drinkers (P = 0.04).
Cardiovascular	Cardiometabolic Syndrome [34]	Men and women	Positive outcome	Average of 2 glasses of red wine per day (women were lower than this average and men were higher than this average)	Moderate red wine consumption was associated with a reduced risk of developing metabolic syndrome.	In this study of 3897 elderly men and women (average age 66) at high cardiovascular risk, moderate red wine consumption was associated with a reduced risk of developing metabolic syndrome versus non-drinkers (OR = 0.56 (0.45-0.68) P < 0.001) as well as the major parameters for metabolic syndrome (abnormal waist circumference OR = 0.59 (0.46-0.77) P < 0.001; Low HDL OR = 0.42 (0.32-0.53) P < 0.001; High BP OR = 0.28 (0.17-0.45) P < 0.001; High Fasting Plasma Glucose OR = 0.67 (0.54-0.82) P < 0.001).
Cardiovascular	Cardiometabolic Syndrome [35]	Men and women	Positive outcome	1 glass of wine (red or white) per day for women and 2 glasses per day for men	Moderate wine consumption was associated with a decrease in the odds of developing metabolic syndrome.	In this study of 15,905 Hispanic/Latino men and women (age 18 to 74), moderate wine consumption was associated with a decrease in the odds of developing metabolic syndrome versus non-drinkers (OR = 0.43 (0.21-0.87)). There was no separate analysis for red wine.
Cardiovascular	Cardiometabolic Syndrome [36]	Men and women	Positive outcome	1 to 1.5 glasses of wine (red or white) per day	Moderate wine consumption was associated with a decrease in the odds of developing metabolic syndrome.	In this study of 64,046 men and women (age 18 to 80), moderate wine consumption was associated with a decrease in the odds of developing metabolic syndrome versus non-drinkers (OR = 0.72 (0.68-0.84) P < 0.001). There was no separate analysis for red wine.
Cardiovascular	Cardiometabolic Syndrome [37]	Men and Women	Positive outcome	> 1 glass of wine (red or white) per day	Moderate wine consumption was associated with a decrease in the odds of developing metabolic syndrome.	In this study of 4510 men and women (average age 52), moderate wine consumption was associated with a decrease in the odds of developing metabolic syndrome versus non-drinkers (OR = 0.32 (0.14-0.73)). There was no separate analysis for red wine.
Cardiovascular	Coronary Heart Disease (CHD) in women with hypertension [38]	Women	Positive outcome	1 to 2 glasses of red wine per day	Moderate red wine consumption was associated with a lower risk of CHD.	In this study of 10,530 hypertensive women followed over 9.4 years, moderate red wine consumption was associated with a lower risk of CHD versus non-drinkers (HR = 0.71 (0.43-1.16) P-trend = 0.032).
Cardiovascular	Central Adiposity as a risk factor for Coronary Heart Disease (CHD) [39]	Men and women	Positive outcome	Average of 2 glasses of wine (red or white) per day for women and 3 for men.	Moderate daily wine consumption was associated with smaller abdominal heights (decreased central adiposity).	In this study of 2343 men and women (age 35 to 79), moderate daily wine consumption was associated with smaller abdominal heights (decreased central adiposity) versus non-drinkers (decrease of 5% in women (P<0.05) and a decrease of 3.5% in men (P<0.05). There was no separate analysis for red wine.
Cardiovascular	Atrial Fibrillation (AF) [40]	Men and women	Neutral outcome	Up to 1 glass of red wine per day for women and up to 2 glasses per day for men	Moderate red wine consumption in a high cardiovascular-risk population was not associated with an increased incidence of AF.	In this study of 6527 men and women (average age 67) at high risk for cardiovascular disease with a mean follow-up of 4.4 years, moderate red wine consumption was not associated with AF incidence versus non-drinkers (HR = 1.09 (0.79-1.51)).
Cardiovascular	Heart Failure (HF), Quality of Life (QOL), and Depression [41]	Men and women	Neutral outcome	1 to 2 glasses of wine (red or white) per day	Moderate wine consumption was not associated with HF but did demonstrate statistical significance for improved QOL and less depression.	In this study of 6973 men and women with HF (average age 67) followed for ~4 years, moderate wine consumption versus non-drinkers was not associated with HF (all-cause death or hospitalization for CV causes HR = 1.04 (0.92-1.18) P-trend = 0.64), but did demonstrate statistical significance for improved QOL (P < 0.0001) and less depression (P = 0.01) vs non-drinkers. There was no separate analysis for red wine.
Cardiovascular	Hypertension (HTN) [42]	Women	Positive outcome	0.75 glass of red wine per day	Moderate intake of red wine was associated with a decreased risk of developing HTN.	In this study of 28848 women (average age 54), moderate intake of red wine was associated with a decreased risk of developing HTN versus non-drinkers (RR=0.73 (0.52-0.99)). NOTE: the authors also assessed men from a different database which had no beverage-specific data, so no wine or red wine analysis was performed for men, only for women.
Cardiovascular	Acute Myocardial Infarction (AMI) [43]	Men and women	Positive outcome	Average of 1.5 glasses of red wine per day	Moderate intake of red wine was associated with a lower risk of nonfatal myocardial infarction (MI).	In this small case-control study of 171 cases of AMI in men and women (average age 62), moderate intake of red wine was associated with a lower risk of nonfatal MI versus non-drinkers (OR = 0.38 (0.15-1.00) P = 0.05).
Cardiovascular	Myocardial Infarction (MI), Stroke, or Cardiovascular (CV) mortality [44]	Men and women	Neutral outcome	1 glass of red wine per day	Moderate red wine consumption was not associated with MI, stroke, or CV mortality.	In this study of 14,651 men and women (average age 42) followed for 14 years, only 127 events occurred, underpowering the study to find any of the 7 endpoints reaching statistical significance. Versus non-drinkers, regarding MI or stroke, moderate red wine consumption (HR of 0.75 (0.49-1.1)) just missed statistical significance; CV mortality (HR of 0.9 (0.45-1.9)) was not statistically significant.
Cardiovascular	Myocardial Infarction (MI) [45]	Men	Neutral outcome	> 1 glass of red wine per day	Moderate intake of red wine was not associated with a risk of MI.	In this study of 38,077 men (age 40 to 75) who were free of cardiovascular disease or cancer, followed for 12 years, 1418 MIs occurred (only 8 in the red wine group), moderate intake of red wine was not associated with a risk of MI versus non-drinkers (RR = 0.64 (0.32-1.29) P = 0.34). Most men in the study drank beer or liquor, consequently, the red wine group was undersized to demonstrate statistical significance.
Cardiovascular	Ischemic Stroke [46]	Men	Positive outcome	1 glass of red wine per day	Red wine consumption was inversely associated with ischemic stroke risk.	In this study of 38,156 men (average age 54) followed for 14 years, 412 cases of incident ischemic stroke were documented. Compared with non-drinkers, red wine consumption was inversely associated with risk in a graded manner (RR = 0.77 (0.61-0.98) P-trend = 0.02).
Dementia	Cognitive Decline [47]	Men and women	Positive outcome	1 glass of red wine per day for women and 2 glasses per day for men.	Moderate red wine consumption was associated with less decline in Global Cognitive Function.	In this study of 2613 men and women (average age 56) followed for 5 years, moderate red wine consumption was associated with less decline in Global Cognitive Function vs non-drinkers (red wine decline -0.03 versus non-drinkers decline of -0.11, P < 0.05).
Dementia	Decline in patients with Alzheimer's Disease (AD) [48]	Men and women	Positive outcome	1 glass of wine (red or white) per day	Moderate wine consumption was not associated with a decline in patients with AD.	In this study of 360 men and women (average age 74) with AD followed for ~5 years, moderate consumption of wine was not associated with a decline versus non-drinkers (Beta coefficient = 0.015, P = 0.826). There was no separate analysis for red wine.
Dementia	Dementia [49]	Women	Positive outcome	1 glass of wine (red or white) per day	Moderate, daily wine consumption provided protective effects against dementia versus beer and spirits, which had the opposite effect on dementia.	In this study of 1458 women followed for 34 years, moderate, daily wine consumption provided protective effects against dementia (HR = 0.32 (0.12-0.81)) versus beer and spirits, which had the opposite effect on dementia. There was no separate analysis for red wine.
Dementia	Dementia [50]	Men and women	Positive outcome	1 glass of wine (red or white) per day	Moderate consumption of wine was associated with a decrease in dementia risk versus light wine drinkers. NOTE: Non-drinkers had more dementia than light wine drinkers, but the authors chose to use light drinkers as the reference.	In this study of 12,326 men and women, followed for 43 years, moderate consumption of wine was associated with a 2% decrease in dementia risk for each 1 gm of alcohol from wine versus light wine drinkers (HR = 0.98 (0.97-0.99) P = 0.003). NOTE: non-drinkers had statistically significantly more dementia than light wine drinkers, but the authors chose to use light drinkers as the reference, which reduced the magnitude of the benefit of moderate drinking. The authors stated that "drinking wine appears to reduce the risk of dementia except at high amounts." There was no separate analysis for red wine.
Dementia	Overall Dementia and Alzheimer's Dementia (AD) [51]	Men and women	Positive outcome	1 to 2 glasses of wine (red or white) per day	Moderate wine consumption was associated with a decreased risk of overall dementia and AD.	In this study of 3202 men and women (age 75+) without dementia, followed for ~3 years, moderate wine consumption was associated with a decreased risk of overall dementia (HR = 0.66 (0.47-0.93) P = 0.017) and AD (HR = 0.61 (0.38-0.97) P = 0.035) versus non-drinkers. There was no separate analysis for red wine.
Eye	Age-related Macular Degeneration (AMD) [52]	Men and women	Neutral outcome	< 1.5 glasses of wine (red or white) per day	Moderate wine consumption was not associated with AMD.	In this study of 20,963 men and women (age 40 to 69) followed for 11+ years, moderate wine consumption was not associated with early AMD (OR = 1.02 (0.92-1.14)). There was no separate analysis for red wine.
Gastrointestinal	Barrett's Esophagus (BE) and Erosive Esophagitis (EE) [53]	Men and women	Neutral outcome	About 1 glass of red wine per day	Moderate red wine consumption trended toward a reduced risk of BE and EE.	This is a small (n=339 BE, n=462 EE), underpowered study that demonstrated trends, but cases were too low to show statistical significance. Results showed a U-shaped trend of reduced BE or EE risk associated with moderate red wine consumption.
Infections	COVID-19 [54]	Men and women	Positive outcome	> 1 glass of red wine per day	Moderate red wine consumption was associated with a lowered risk of developing COVID-19 by 17%.	In this study of 473,957 men and women (average age 70), moderate red wine consumption compared with non-drinkers was associated with a lowered risk of developing COVID-19 by 17% (OR = 0.83 (0.78-0.88) P < 0.001).
Infections	Common Cold [55]	Men and women	Positive outcome	> 1 glass of red wine per day	Moderate red wine consumption was associated with a reduced risk of developing a common cold.	In this study of 4272 men and women (age 21 to 69), moderate red wine consumption was associated with a reduced risk of developing a common cold versus non-drinkers (RR = 0.39 (0.18-0.87)).
Kidney	Kidney Stones [56]	Men	Positive outcome	1.5 glasses of wine (red or white) per day	Moderate wine consumption was associated with a decreased risk of kidney stone formation by 39%.	In this study of 45,289 men (age 40 to 75) followed over 6 years, moderate consumption of wine versus non-drinkers was associated with a decreased risk of kidney stone formation by 39% (RR = 0.61 (0.42-0.9) P = 0.02). There was no separate analysis for red wine.
Liver	Cirrhosis [57]	Men and women	Positive outcome	1 to 2.5 glasses of wine (red or white) per day	Moderate wine consumption was associated with a lower risk of cirrhosis versus very light drinkers and also versus beer or spirits drinkers.	In this study of 30,630 men and women (age 52) followed for 417,325 person-years, moderate wine drinkers were associated with a lower risk of cirrhosis versus very light drinkers (< 1 drink per week) (wine up to 1 drink/day RR = 0.5 (0.4-0.7); wine up to 2.5 drink/day RR = 0.6 (0.3-1.0)). At all levels of alcohol consumption, wine had a lower RR of cirrhosis versus beer and spirits. There was no separate analysis for red wine.
Mortality	All-cause Mortality, Coronary Heart Disease (CHD) mortality, and Cancer mortality [58]	Men and women	Positive outcome	1 to 2.5 glasses of wine (red or white) per day	Moderate wine consumption was associated with a lower relative risk for death from all-cause mortality, from CHD, and from cancer.	In this study of 24,523 men and women (age 20 to 98) with follow-up for 257,859 person-years, moderate consumption of wine was associated with a reduced risk of death compared to non-drinkers from all-causes (RR=0.76 (0.67-0.86) P<0.001), from CHD (RR=0.64 (0.48-0.84) P = 0.007), and from cancer (RR=0.78 (0.64-0.96) P = 0.004). There was no separate analysis for red wine.
Mortality	All-cause Mortality [59]	Men and women	Positive outcome	0.5 to 1.5 glasses of red wine per day for women and 0.5 to 3.5 glasses per day for men	Moderate red wine consumption was associated with lower mortality.	In this study of 18,394 men and women (average age 39) followed up to 12 years, moderate red wine consumption was associated with lower mortality versus non-drinkers (HR = 0.55 (0.35-0.88) p = 0.012).
Mortality	All-cause Mortality [60]	Men	Positive outcome	3 to 5.5 glasses of red wine per day	Moderate daily consumption of red wine was associated with the longest life expectancy versus occasional or heavy drinkers.	In this study of 1536 men (age 45 to 64) followed for 30 years, moderate daily consumption of red wine was associated with the longest life expectancy (about 2 extra years) versus occasional or heavy drinkers.
Mortality	All-cause Mortality [61]	Men and women	Positive outcome	1 to < 3 glasses of wine (red or white) per day	Moderate wine consumption was associated with a reduced risk of mortality.	In this study of 802 men and women (age 55 to 65) followed for 20 years, moderate wine consumption was associated with a lower all-cause mortality versus non-drinkers (OR = 0.59 (0.42-0.82) P < 0.01). There was no separate analysis for red wine.
Mortality	All-cause Mortality [62]	Men and women	Positive outcome	1 to 2 glasses of red wine per day	Moderate red wine consumption was associated with a significantly lower mortality.	In this study of 128,934 men and women (average age 41) followed for 20 years, moderate red wine consumption was associated with lower mortality versus non-drinkers (RR = 0.8 (p < 0.001)).
Mortality	All-cause Mortality, Cardiovascular Events, Ischemic Heart Disease (IHD), Cerebrovascular Events [63]	Men and women	Positive outcome	1 to 1.5 glasses of red wine per day	Moderate consumption of red wine was associated with a decrease in all-cause mortality, cerebrovascular events, and IHD.	In this study of 502,635 men and women (age 40 to 69) followed for 7 years, moderate consumption of red wine was associated with a decreased risk of all-cause mortality (HR = 0.81 (0.73-0.89) p
Mortality	Coronary Heart Disease (CHD), Cardiovascular Disease (CVD), or All-cause Mortality [64]	Men	Positive outcome	< 1.5 glasses of wine (red or white) per day	Moderate wine consumption was associated with a reduced risk of mortality from CHD, CVD, and all-cause mortality.	In this study of 1373 men (age > 40) followed for 40 years, moderate consumption of wine was associated with a reduced risk of mortality from CHD, CVD, and all-cause mortality versus non-drinkers (CHD mortality HR = 0.61 (0.41-0.89), CVD mortality HR = 0.68 (0.53-0.86), all-cause HR = 0.73 (0.62-0.87)). Life expectancy was 5 years longer in wine drinkers versus non-drinkers. There was no separate analysis for red wine.
Mortality	All-cause Mortality, Major Cardiovascular Event (MACE), Liver Cirrhosis [65]	Men and women	Positive outcome	Up to 1 glass of red wine per day for women and up to 1.5 glasses per day for men	Moderate red wine consumption was associated with a lower risk of all-cause mortality, MACE, and liver cirrhosis versus beer, cider, or spirits.	In this study of 309,123 men and women (age 38 to 73) followed for 9 years, red wine drinking was associated with a lower risk of all-cause mortality (ACM), MACE, and liver cirrhosis (LC) versus beer, cider, or spirits. Non-drinkers were excluded. Red wine drinkers were the reference point of 1.0 (beer/cider ACM HR = 1.18 (1.1-1.27) P<0.01; MACE HR = 1.16 (1.05-1.27) P<0.01; LC HR = 1.36 (1.06-1.74) P = 0.02) (spirits ACM HR = 1.25 (1.14-1.38) P<0.01; MACE HR = 1.31 (1.15-1.5) P<0.01; LC HR = 1.48 (1.08-2.03) P = 0.01).
Mortality	All-cause Mortality [66]	Men	Positive outcome	< 4 glasses of wine (red or white) per day	Moderate wine consumption was associated with a decrease in all-cause mortality in men with either normal systolic blood pressure (SBP) or in those with elevated SBP.	In this study of 36,583 men (age 40 to 60) with normal baseline SBP followed for up to 21 years, moderate wine consumption was associated with a decrease in all-cause mortality versus non-drinkers in either men with normal SBP or elevated SBP (SBP 158mm RR = 0.77 (0.62-0.96) P < 0.02; SBP 139mm RR = 0.73 (0.58-0.91) P < 0.01; SBP 116mm RR = 0.63 (0.51-0.78) P < 0.001). There was no separate analysis for red wine.
Mortality	All-cause Mortality [67]	Men	Positive outcome	Up to 3.5 glasses of wine (red or white) per day	Moderate wine consumption was associated with a decreased risk of death from all causes.	In this study of 36,250 men (age 40 to 60) followed for up to 18 years, moderate wine consumption was associated with a decreased risk of death from all causes versus non-drinkers (RR = 0.71 (0.63-0.82) P < 0.001). There was no separate analysis for red wine.
Nervous System	Depression [68]	Men and women	Positive outcome	Up to 1 glass of red wine per day	Moderate red wine consumption was associated with a reduced risk of depression.	In this study of 5505 men and women (age 55 to 80) followed up to seven years, moderate red wine consumption was associated with a reduced risk of depression versus non-drinkers (RR = 0.57 (0.36-0.92)).
Nervous System	Multiple Sclerosis (MS) [69]	Men and women	Positive outcome	0.5 to 1 glass of wine (red or white) per day for women and 1 to 1.5 glasses of wine per day for men	Moderate wine consumption was associated with a reduced odds of developing MS.	In this study of 13,626 men and women (average age 35), moderate wine consumption was associated with reduced odds of developing MS versus non-drinkers (OR = 0.7 (0.5-0.8) P = 0.001). There was no separate analysis for red wine.
Nervous System	Amyotrophic Lateral Sclerosis (ALS) [70]	Men and women	Positive outcome	Undefined (red wine glasses/week was part of the data capture, but authors did not include the data in the article)	Red wine consumption was associated with a reduced odds of developing ALS.	In this study of 1557 men and women (average age 64) with ALS compared to matched controls, consumption of red wine was associated with reduced odds of developing ALS versus non-drinkers (OR = 0.83 (0.7-0.97) P = 0.021).
Respiratory	Pulmonary Function [71]	Men and women	Positive outcome	About 1 glass of red wine per day	Moderate red wine consumption was associated with improved pulmonary function.	In this study of 1555 men and women (average age 59), moderate red wine consumption was associated with improved pulmonary function versus non-drinkers (FEV1 regression coefficient = 1.277 (0.008-2.546) p < 0.05; FVC regression coefficient = 1.262 (0.044-2.48) p < 0.05).
Weight	Body Mass Index (BMI) [72]	Men and women	Positive outcome	Up to 1 glass per day of red wine for women and up to 1.5 glasses per day for men	Moderate red wine consumption was associated with a lower BMI.	In this study of 280,183 men and women (average age 56), moderate red wine consumption was associated with a lower BMI versus non-drinkers (BMI association = -0.75 (-0.78 to -0.72) P < 0.001).
Weight	Waist Circumference [73]	Men and women	Positive outcome	1 glass of wine (red or white) per day for women and 1.5 glasses per day for men	Moderate wine consumption was associated with a decreased risk of a major gain in waist circumference.	In this study of 43,543 men and women (age 50 to 64) with a follow-up after five years, moderate wine consumption was associated with a decreased risk of a major gain in waist circumference versus drinkers of < 1 glass per week (women OR = 0.82 (0.7-0.96) P-trend = 0.009; men OR = 0.67 (0.51-0.89) P-trend = 0.005). There was no separate analysis for red wine.
Weight	Overweight or Obese [74]	Women	Positive outcome	> 1 glass of red wine per day	Moderate red wine consumption was associated with a decrease in the risk of becoming overweight or obese.	In this study of 19,220 normal-weight women (average age 39) followed for 13 years, moderate red wine consumption was associated with a decrease in the risk of becoming overweight or obese versus non-drinkers (RR = 0.66 (0.52-0.84) P<0.001).
Weight	Weight gain > 11 pounds [75]	Women	Positive outcome	1 to 2 glasses of wine (red or white) per day	Moderate wine consumption was associated with a lower risk of weight gain.	In this study of 49,324 women (age 27 to 44) followed for eighty years, moderate wine consumption was associated with a lower risk of weight gain (defined as > 11 pounds) versus non-drinkers (OR = 0.92 (0.84-0.99) P-trend=0.01). There was no separate analysis for red wine.
Weight	Weight gain > 4% [76]	Men	Neutral outcome	Up to 2 glasses of wine (red or white) per day	Moderate wine consumption was not associated with weight gain versus non-drinkers.	In this study of 7608 men (age 40 to 59) followed for 5 years, moderate wine consumption was not associated with weight gain (defined as > 4%) versus non-drinkers (OR = 0.96 (0.81-1.12)). There was no separate analysis for red wine.
Weight	Weight changes with long-term alcohol consumption [77]	Men	Neutral outcome	Based on 1 glass increments of wine (red or white) per day	Moderate wine consumption was not associated with weight gain versus non-drinkers.	In this study of 14,971 men (age 40 to 65) followed for up to 24 years, moderate wine consumption was not associated with weight gain versus non-drinkers (increase in weight for every 1 drink per day = 0.16 pounds (-0.04 to 0.36) P = 0.11). There was no separate analysis for red wine.
Weight	Weight and waist circumference changes with long-term alcohol consumption [78]	Men and women	Neutral outcome	1.5 to 3 glasses of wine (red or white) per day	Moderate wine consumption was not associated with weight gain but was associated with a slightly reduced risk of increasing waist circumference versus non-drinkers.	In this study of 5879 men and women (age 40 to 69) followed for ~12 years, moderate wine consumption was not associated with weight gain but was associated with a slightly reduced risk of increasing waist circumference versus non-drinkers. Wine's beta coefficient for weight gain was -0.25 (SE 0.16, P = 0.12) and for waist circumference enlargement was -0.67 (SE 0.23, P = 0.003). There was no separate analysis for red wine.

**Figure 2:**
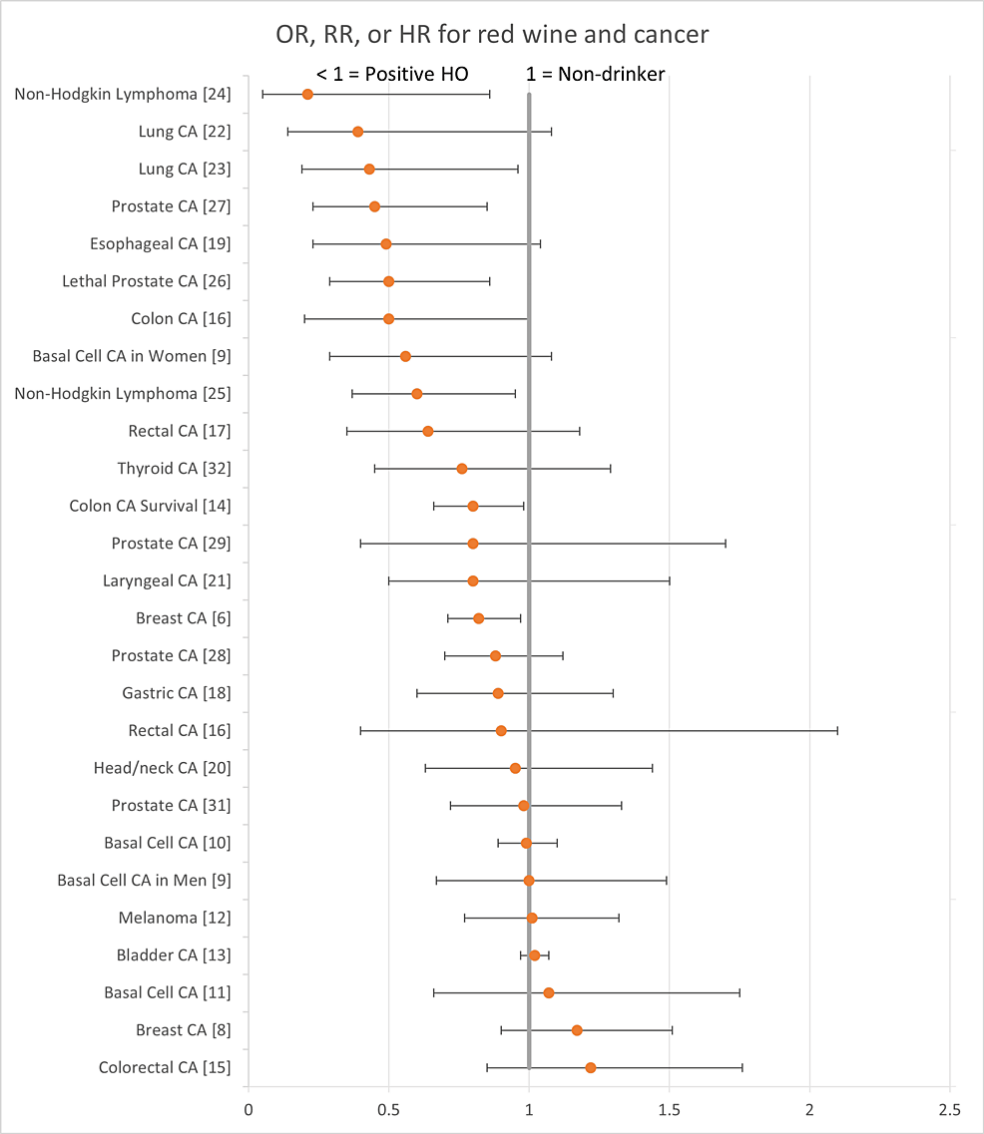
Red wine and cancer Red wine consumption was associated with positive health outcomes for non-Hodgkin lymphoma. Its impact on all other cancers was either neutral or a mix of positive and neutral health outcomes. There were no negative health outcomes. CA: Cancer

**Figure 3:**
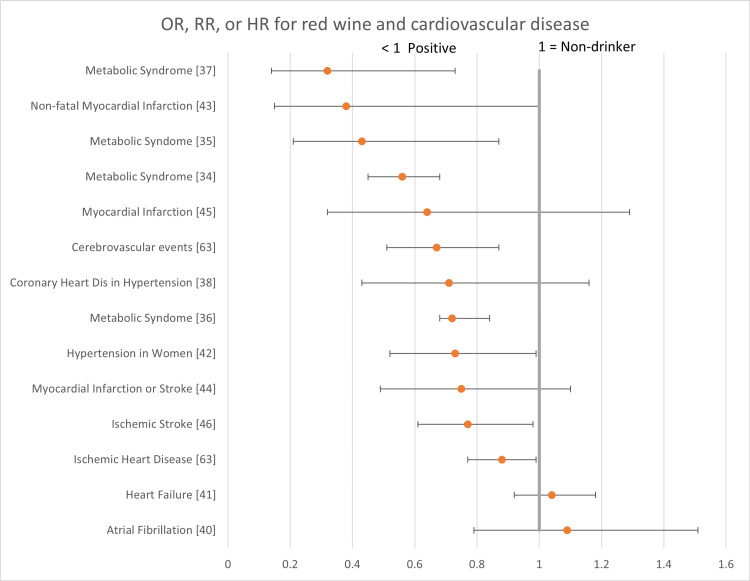
Red wine and cardiovascular disease Red wine consumption was associated with positive health outcomes for metabolic syndrome. Its impact on all other cardiovascular diseases was either neutral or a mix of positive and neutral health outcomes. There were no negative health outcomes.

**Figure 4:**
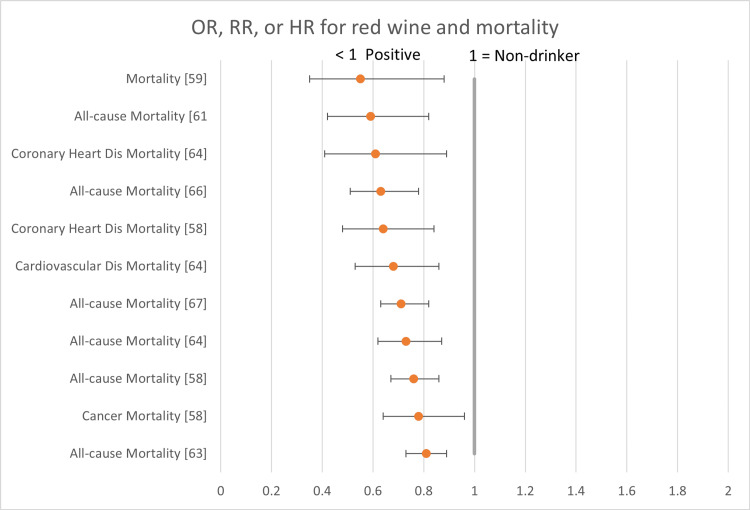
Red wine and mortality Red wine consumption was associated with positive health outcomes in all the mortality studies.

**Figure 5:**
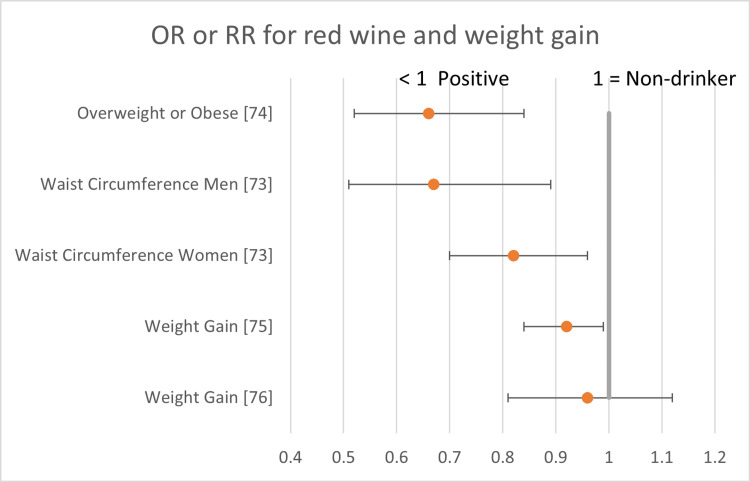
Red wine and weight gain Red wine consumption demonstrated either positive health outcomes or was neutral for weight gain. There were no negative health outcomes.

**Figure 6:**
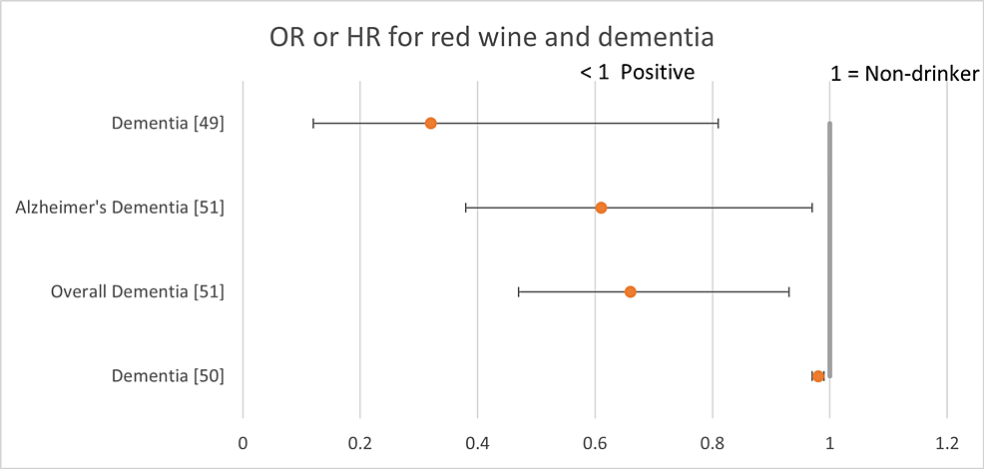
Red wine and dementia Red wine consumption was associated with positive health outcomes in all the dementia studies.

**Figure 7:**
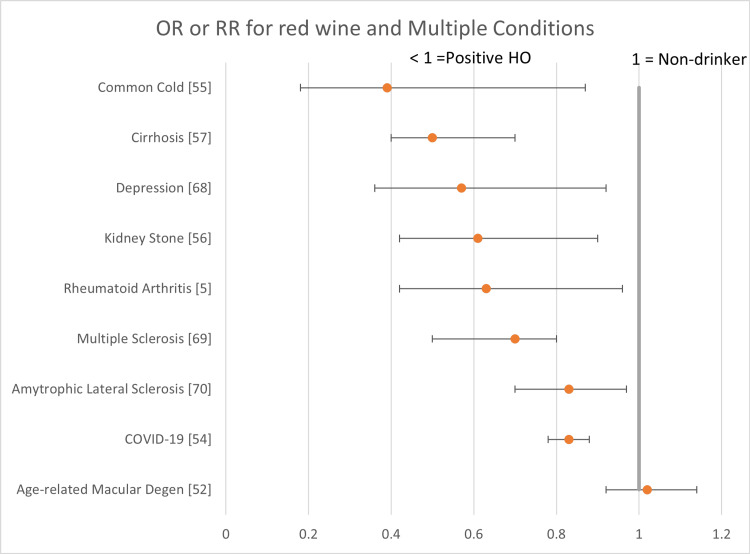
Red wine and multiple conditions Red wine consumption was associated with positive health outcomes across multiple conditions. There were no negative health outcomes.

The following forest plots (Figures 2-7) show the data for studies that contained an OR, RR, or HR versus non-drinkers. The reference point for non-drinkers in the forest plots is 1.0. An OR, RR, or HR < 1.0 indicates a positive health outcome for red wine. Some studies had more than one endpoint, as shown in the forest plots. Studies that did not have an OR, RR, or HR (e.g., beta coefficient) are not shown in the forest plots but are included in the full summary given above in Table 2.

Discussion

This systematic review intends to highlight the currently available data about the health outcomes of moderate daily consumption of red wine (as defined above). This allows readers to make informed decisions about red wine consumption. While these studies provide us with this necessary information, there are, of course, limitations to any study, which can make the interpretation of the results quite challenging. These limitations include the type of red wine consumed, data capture on volume and frequency of consumption, lack of multivariate data analysis, absence of a standardized definition of a “red wine drinker”, and non-standardized study methodology. It is also important to remember that an improvement in an OR, RR, or HR must be tempered by the fact that this equates to a rather small change in absolute risk.

There is scientific evidence that polyphenols in wine provide antioxidant protection, decrease platelet aggregation, increase endothelial function, and attenuate inflammatory responses, which leads to possible cardioprotective, neuroprotective, and chemopreventive effects [2]. Wine polyphenols mainly come from the skins and seeds of grapes, with more in red wine than in white wine. This difference will influence the outcomes of red wine on health in a study [2]. The increased cost of more expensive, premium, complex wine is driven by multiple factors, not the least of which is the extended time spent macerating the skins and aging the wines in oak to extract as much of the polyphenols as possible. Contrast this with mass-produced, inexpensive, simple red wine, which spends less time in maceration or oak aging, hence keeping the costs down, but also contains fewer polyphenols. As reported at the Unified Wine and Grape Symposium 2023, wines under $8 make up 73% of the US wine market, and wines over $15 make up only 13%. [79]. Since current studies lack a mechanism to distinguish between complex and simple red wine, it stands to reason that study participants who declare red wine consumption may not be drinking complex red wine, therefore consuming fewer polyphenols. Despite this, the studies still demonstrate benefits or neutrality regarding health outcomes with moderate red wine consumption.

Data capture for alcohol research has evolved over the years to add more parameters that allow for the demonstration of key differences, such as the effects of red wine versus other alcohols. Red wine is often not the focus of the study, which makes data interpretation challenging. A separate red wine analysis was performed in 41 of the studies included in this review. The remaining 33 studies performed a wine analysis that contained both red and white wine. Combining the results of red and white wine may alter the health outcomes in a study since red wine has been shown to demonstrate more health benefits than white wine due to the larger amount of polyphenols it contains [2]. Table 2 identifies the studies that did not perform a separate red wine analysis.

Comprehensive health questionnaires were used for many of the studies in this review. Consequently, investigators have found that there can be differences in baseline health status that exist for the participants that are associated with the preferred type of alcohol consumed. There are claims in some studies that wine drinkers versus other alcohol drinkers are healthier to begin with. For example, they do more exercise, eat healthier, avoid smoking, are more often light to moderate drinkers, have higher education and income levels, and have access to medical care) [80,81]. While most studies adjust for these health differences using a multivariate analysis, some do not. When performing a meta-analysis, it is imperative to avoid a broad application of a "numeric factor" used to adjust all the results. This factor should only be applied to studies that don't already use a multivariate adjustment. Otherwise, these factors may negate legitimate differences (e.g., potential benefits) found in these studies. 

There are several limitations in the alcohol data captured in the studies that make it challenging to determine the correct number of drinks per day that is statistically significant. Health questionnaires that are used in these studies to capture alcohol consumption often have forced ranges (i.e., 1 to 7 drinks per week, 8 to 14, 15 to 21, etc.) and are completed by participants retrospectively (e.g., annually), which could lead to poor recall. Additionally, if the weekly amount is consumed over seven days versus all on the weekend, this could make the difference between moderate consumption and binge drinking but would not be differentiated on the questionnaire and could influence the health outcomes. Some questionnaires (e.g., the National Institutes of Health (NIH) Food Frequency Questionnaire [82]) have more detailed categories to allow a better understanding of the number of drinks and the frequency but are still completed retrospectively. Adding to the lack of accuracy in reporting, the definition of a "drink" is not standard worldwide, nor are the sizes of glassware, so what a participant reports as a drink in the study is uncertain. Since underreporting of alcohol consumption is common, with up to 60% underreporting [83,84], the range for moderate consumption reported within a study is lower than actual consumption and should technically be adjusted upwards by a reasonable factor to be accurate. All these limitations make it difficult to truly assess health outcomes based on moderate consumption levels, and this is important since health outcomes from red wine tend to follow a J-shaped curve [85], where moderate consumption may offer benefits over abstinence. However, beyond moderation, benefits are potentially lost, and health outcomes become negative with increasing consumption.

Further, when a study participant reports that they drink wine, it is uncommon for the researchers to analyze the data based on the percentage of wine a person drinks versus beer or spirits. Rarely does someone exclusively drink wine (or red wine) versus some blend of wine, beer, and spirits. Consequently, what constitutes a "red wine drinker" needs to be made clear in each study.

Across the 74 studies in this review, the study design varied, making it difficult to compare results from one study to the next. Developing standards for conducting these types of studies would enhance the ability to interpret the data and provide more definitive answers regarding the outcomes of health. Ideally, randomized controlled trials (RCTs) are needed to help make this determination. However, this search only revealed one RCT [33], which was positive for red wine in cardiometabolic syndrome, which is included in this review. Long-term RCTs on this subject are difficult to design from an ethical standpoint, forcing subjects to drink alcohol daily for long periods, and even more challenging to conduct due to issues with long-term participant compliance.

Searching the literature for the term "health outcomes" may have biased this search because the search term could imply positive outcomes versus all outcomes (i.e., positive, neutral, or negative). Consequently, for this reason, and potentially others, there may be additional studies that should have met the criteria but were not identified in this search. While limitations have been noted in the studies reviewed for this article, the hope for future research is to develop data collection standards for a more accurate analysis of the effects of red wine consumption on health outcomes.

## Conclusions

From this systematic review of the literature, there is no evidence of an association between moderate red wine consumption and negative health outcomes. Across the various outcomes assessed, a beneficial effect of moderate red wine consumption was consistently seen for mortality and dementia, along with certain cancers (i.e., non-Hodgkin lymphoma) and cardiovascular conditions (i.e., metabolic syndrome). For other health outcomes, the association was neutral, i.e., neither harmful nor beneficial, or a mix of positive and neutral. Even if moderate consumption of red wine is only neutral on health outcomes, the potential positive psychosocial benefits should not be ignored.

While this review does not intend to encourage red wine consumption for health outcomes, it does hope to prevent the discouragement of moderate red wine consumption based on broad conclusions drawn from data pooled across all types of alcohol. It must be emphasized that these conclusions are based on moderate consumption levels since health outcomes from red wine tend to follow a J-shaped curve where moderate consumption may offer benefits over abstinence. Beyond-moderate consumption of red wine leads to a potential loss of benefits, and health outcomes become negative with increased consumption.
